# Targeting the 3C Protease of Hepatitis A Virus Subgenotype IB: Virtual Screening and Identification of Potent Lead Candidates

**DOI:** 10.3390/microorganisms14091987

**Published:** 2026-09-08

**Authors:** Tatsuo Kanda, Reina Sasaki-Tanaka, Hiroaki Okamoto, Shuji Terai, Cole D. Cwiklowski, Kalyan C. Nagulapalli Venkata

**Affiliations:** 1 Division of Gastroenterology and Hepatology, Uonuma Institute of Community Medicine, Niigata University Medical and Dental Hospital, 4132 Urasa, Minamiuonuma 949-7302, Japan; 2 Division of Gastroenterology and Hepatology, Niigata University Graduate School of Medicine, Dentistry and Health Sciences (Medicine), Niigata University, Niigata 951-8510, Japan; reina_sasaki_0925@yahoo.co.jp (R.S.-T.);; 3 Division of Virology, Department of Infection and Immunity, Jichi Medical University School of Medicine, Shimotsuke 329-0498, Japan; hokamoto@jichi.ac.jp; 4 Department of Medicinal Chemistry, University of Health Sciences and Pharmacy, St. Louis, MO 63010, USA; 5 Department of Pharmaceutical and Administrative Sciences, Saint Louis College of Pharmacy, University of Health Sciences and Pharmacy, St. Louis, MO 63010, USA

**Keywords:** drug discovery, HAV 3C protease inhibitors, hepatitis A virus, in silico, HA11-1299

## Abstract

Hepatitis A virus (HAV) infection remains a global public health concern in both developing and developed countries. In the present study, we identified anti-HAV drugs, using AutoDock Vina Modeling software, and evaluated the compounds in vitro. Following cytotoxicity for Huh7 cells, 5 out of 10 compounds were selected. We evaluated effective HAV 3C protease inhibitors with activity against both HAV genotype IB HM175/18f and HAV genotype IIIA HA11-1299-infected human hepatoma cells. Among the five compounds, we identified only one (**KCN-A-12**), which had an inhibitory effect on both HAV genotype IB HM175/18f and HAV genotype IIIA HA11-1299 replication in human hepatoma Huh7 cells. **KCN-A-12** has more effective inhibitory effects on the replication of HAV genotype IB HM175/18f than that of HAV genotype IIIA HA11-1299. This difference may be attributable to the fact that our discovery system depends on crystal structures from HAV 3C protease based on the HAV genotype IB HM175 strain. In conclusion, we observed that **KCN-A-12** was able to inhibit HAV replication. In silico screening for HAV 3C protease inhibitors may be useful for further discovery of anti-HAV drugs.

## 1. Introduction

In developing and developed countries, hepatitis A virus (HAV) infection is a life-threatening disease [1]. In 2021, the estimated global incidence of and deaths from hepatitis A were 56,100,000 and 27,400 persons/year, respectively; the global incidence of and deaths from foodborne hepatitis A were 20,000,000 and 11,900 persons/year, respectively. The proportion of illnesses that are foodborne is about 0.356 [1]. We also reported a hepatitis A outbreak associated with a revolving sushi bar in Japan [2]. Thus, HAV infection is a serious foodborne non-diarrheal enteric disease.

Recently, HAV infection has been linked to “person-to-person contact” or sexually transmitted diseases [3]. In Japan, a male-dominant hepatitis A outbreak has been observed among men who have sex with men (MSM), both among those who have been infected with HIV and among those who have not [4]. MSM have a high risk for HAV infection [5].

To prevent HAV infection, HAV vaccines should be administered. In particular, travelers who go to areas endemic for hepatitis A should be vaccinated [6]. In Korea, similarly to the situation in Japan, young adults seem not to have a protective antibody against HAV, although a universal vaccination program for newborns has been underway since 2015 [7]. A total of 10,083 confirmed cases were reported in seven months in 2019 according to the Korean Centers for Disease Control and Prevention (KCDC). Therefore, it is important to develop anti-HAV drugs and to disseminate HAV vaccines.

We previously reported the in silico screening of anti-HAV compounds targeting the 3C protease enzyme using the Schrödinger Modeling software from an antiviral library of 25,000 compounds and identified HAV 3C protease inhibitor Z10325150 [8]. Artificial intelligence could identify anti-HAV drugs more effectively [8,9]. In the present study, we developed anti-HAV drugs, using the AutoDock Vina Modeling software, and evaluated them in vitro. Compared to our previous study [8], this work broadened both the chemical space and screening strategy. Instead of relying mainly on docking scores from an antiviral-focused Enamine library, we screened a larger and more diverse ZINC-based library [10]. We prioritized candidates using a multiparameter approach that considered active-site interactions, ligand efficiency, scaffold diversity, physicochemical properties, molecular size, and commercial availability.

## 2. Materials and Methods

### 2.1. Software, Database, and Molecular Docking

AutoDock Vina v1.2.5 [11] was used to perform molecular docking. The crystal structure of hepatitis A virus (HAV) 3C protease was obtained from the Protein Data Bank (PDB ID: 2CXV) [12]. A virtual screening library comprising commercially available small molecules was compiled from the ZINC database and commercially sourced from Combi-Blocks (San Diego, CA, USA). Chemical structures were converted to three-dimensional conformations with Avogadro 2 and further processed using Open Babel v3.1.1. Ligand preparation was performed using Meeko v0.7.1 and RDKit v2025.03.6 to generate docking-ready structures in PDBQT format. Lipophilicity values (cLogP) were calculated using ChemDraw Prime v23.1.1.3.

### 2.2. Receptor Preparation

The search grid was positioned over the substrate-binding groove between the two β-barrel domains of the protease, encompassing the catalytic residues His44, Asp84, and Cys172, the oxyanion-hole region, and adjacent substrate-recognition residues. The grid box was centered at x = −1.344, y = −4.165, and z = 31.408, with dimensions of 22 × 22 × 22 Å. Docking utilized an exhaustiveness value of 32, retained 20 poses per ligand, and applied an energy range of 4 kcal mol^−1^. All ligands were docked against the same receptor using identical grid coordinates, box dimensions, and search parameters. For each compound, the most favorable Vina score among the generated poses was recorded. Batch docking was automated using a custom Python v2.7.11. script that processed the ligand set sequentially under these conditions.

### 2.3. Selection of Compounds for Antiviral Activity Evaluation

**Figure 1:**
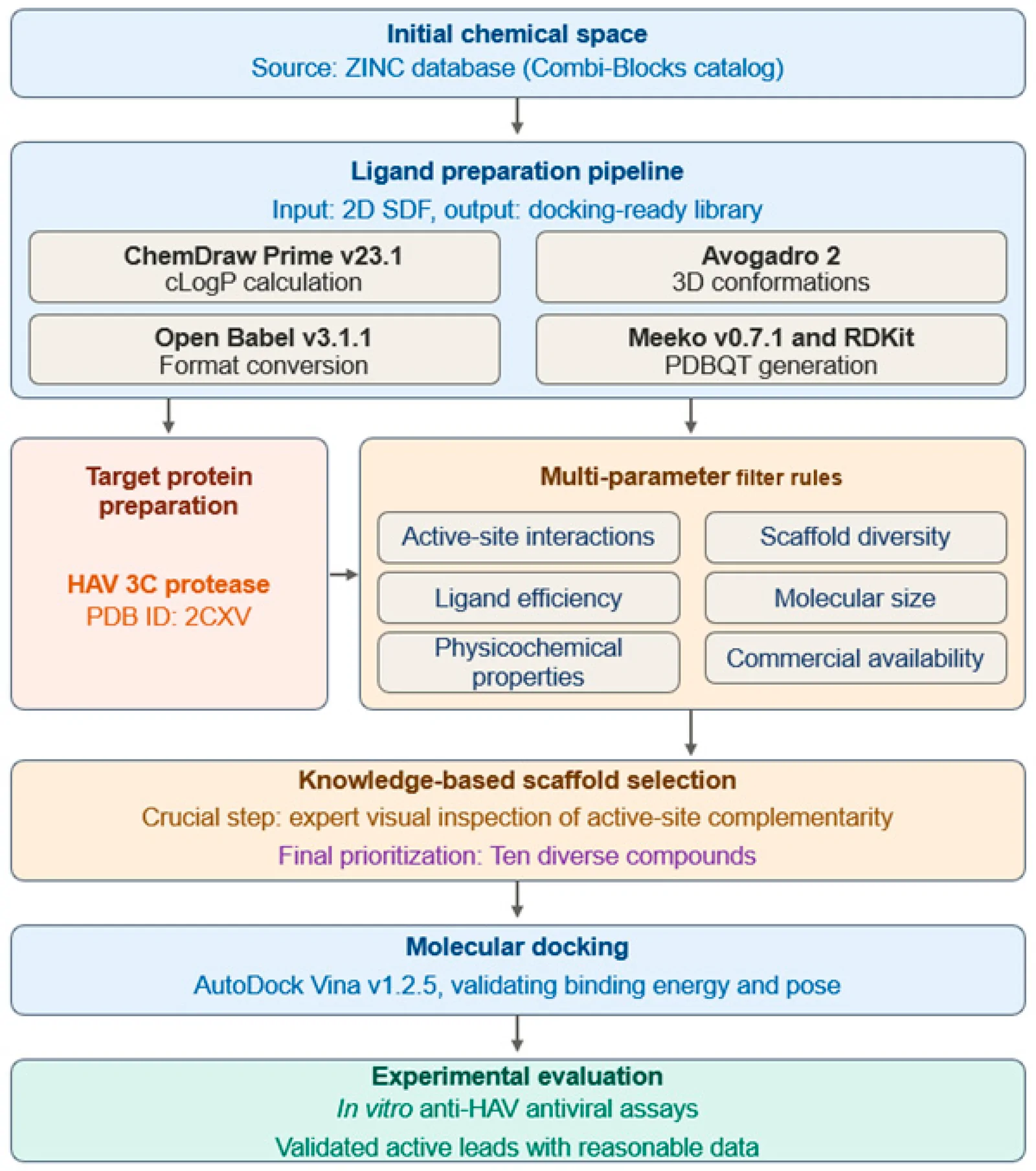
Multi-parameter approach to in silico molecular modeling.

We commercially sourced prioritized compounds from Combi-Blocks (San Diego, CA, USA) and carried them forward for biological evaluation using a multiparameter approach rather than relying solely on docking scores (Figure 1).

**Figure 2:**
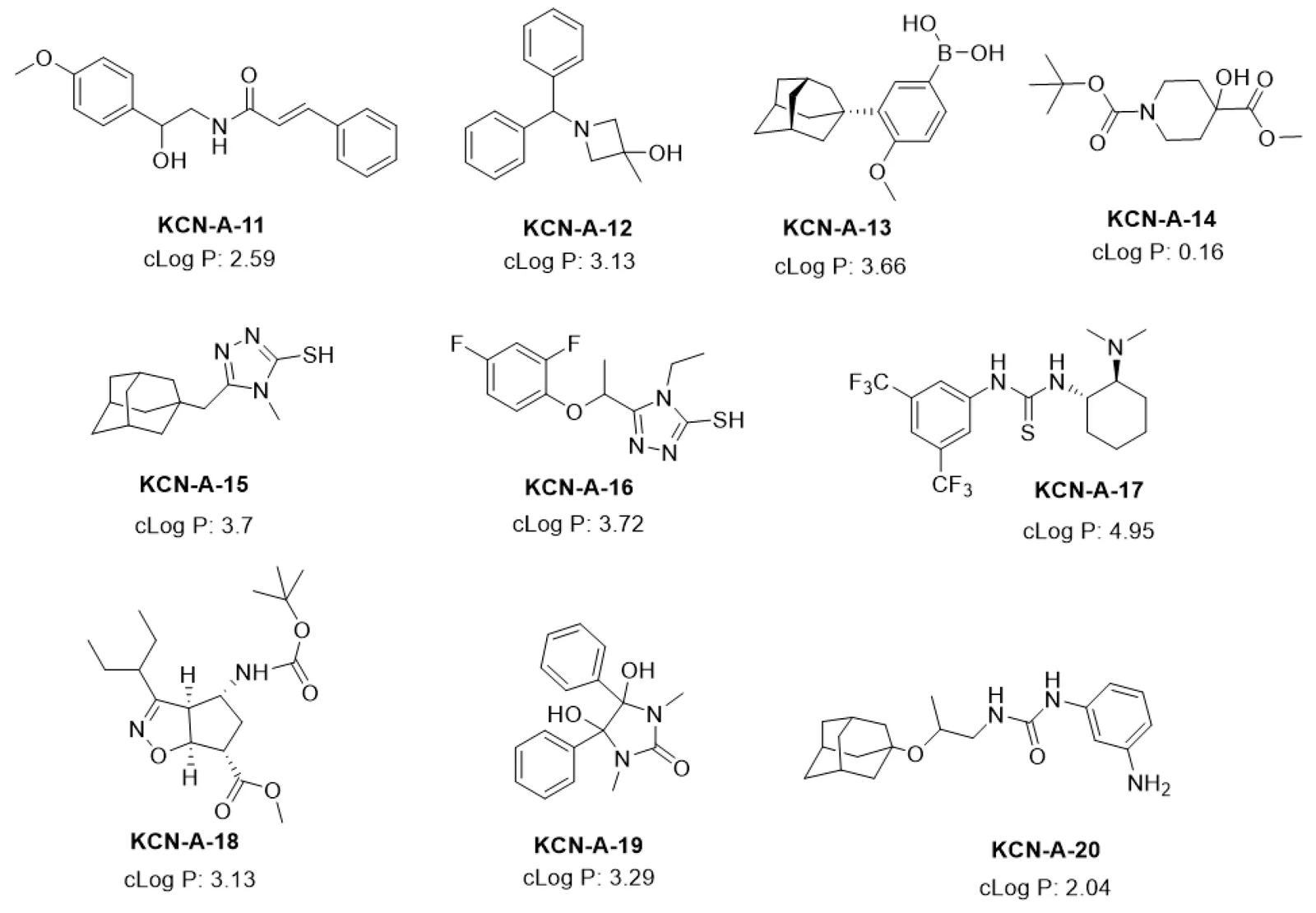
Chemical structures of **KCN-A-11** to **KCN-A-20** selected for antiviral activity evaluation.

Selection criteria included the ability of each ligand to adopt a plausible orientation within the catalytic region; predicted interactions with His44, Asp84, Cys172, the oxyanion-hole region, and neighboring residues; scaffold diversity; calculated physicochemical properties such as cLogP; and commercial availability. Ten compounds, designated **KCN-A-11** to **KCN-A-20** (Figure 2), were procured and assessed for anti-HAV activity and cytotoxicity.

### 2.4. Analysis of Predicted Binding Modes and Ligand Efficiency

Docked complexes were analyzed and superimposed using UCSF ChimeraX v1.12rc. Protein–ligand contacts were characterized with BIOVIA Discovery Studio Visualizer 2025 (v25.1.0.24284) to assess hydrogen bonding, hydrophobic and π–alkyl contacts, π–sulfur interactions, and van der Waals contacts. To account for the influence of molecular size on empirical docking scores, ligand efficiency (LE) was calculated for each compound as LE = −S/N, where S represents the best AutoDock Vina score and N is the number of non-hydrogen atoms [13], enabling size-independent comparison across the series.

### 2.5. Cell Lines and Reagents

Human hepatoma cell line Huh7, which was kindly provided by Prof. Bartenschlager [14], was kept in Roswell Park Memorial Institute medium (RPMI; Sigma-Aldrich, St. Louis, MO, USA) containing 10% heat-inactivated fetal bovine serum (FBS; Sigma-Aldrich), 100 units/mL penicillin, and 100 μg/mL streptomycin (Sigma-Aldrich) under a 5% CO2 atmosphere at 37 °C. HAV HM175 18f genotype IB and HAV HA11-1299 genotype IIIA were used for HAV infection in the present study [15]. HAV HM175/18f genotype IB was provided by Prof. Stanley M. Lemon (University of North Carolina at Chapel Hill, Chapel Hill, NC, USA) and Dr. Asuka Hirai-Yuki (National Institute of Infectious Diseases, Tokyo, Japan).

### 2.6. Cell Viability Assays

For the evaluation of cell viability, dimethylthiazol carboxymethoxyphenyl sulfophenyl tetrazolium (MTS) assays were performed using the CellTiter 96 Aqueous One-Solution cell proliferation assay (Promega, Madison, WI, USA). The MTS assay was performed on a Bio-Rad iMark microplate reader (Bio-Rad, Hercules, CA, USA) at a 490 nm wavelength [15]. Trypan blue dye exclusion assays were also performed with 0.4% trypan blue solution (Sigma-Aldrich), and both live and dead cells were counted under phase contrast microscopy (CKX53, Olympus, Tokyo, Japan) [16].

### 2.7. Half-Maximal Inhibitory Concentration (IC_50_)

IC_50_ indicates the concentration of each compound that produces 50% of the maximal inhibitory effects on HAV replication. IC_50_ was calculated using a previously described formula [17].

### 2.8. Infection of Huh7 Cells with HAV Genotype IB HM175/18f or HAV Genotype IIIA HA11-1299

HAV genotype IB HM175/18f and HAV genotype IIIA HA11-1299 were used for HAV infection in the present study. Before 24 h of infection, Huh7 cells were seeded at a density of 5 × 10^5^ cells/well on 6-well plates (VIOLAMO, AS ONE, Osaka, Japan). Cells were washed twice with phosphate-buffered saline (PBS) (FUJIFILM Wako Chemicals, Osaka, Japan) and infected with HAV genotype IB HM175/18f or HAV genotype IIIA HA11-1299 at a multiplicity of infection (MOI) of 0.01 in serum-free medium. HAV inoculum was incubated with hepatocytes for 6 h, and we added 1 mL of medium containing 2% FBS. After 24 h of incubation, cells were washed once with PBS, followed by the addition of 1 mL of RPMI containing 5% FBS [15]. Then, several concentrations of compounds were added to HAV-infected Huh7 cells according to the results of cell viability assays. After 72 h of infection, cellular RNA was extracted using the RNeasy Mini Kit (Qiagen, Hilden, Germany), and HAV RNA levels were determined using real-time RT-PCR

### 2.9. Extraction of Cellular RNA, cDNA Synthesis, and Real-Time RT-PCR

Total cellular RNA was extracted by the QIAshredder (Qiagen) and RNeasy Mini Kit (Qiagen), according to the manufacturer’s instructions. Then, cDNA synthesis was performed using PrimeScript RT reagent (Perfect Real Time, TaKaRa Bio, Kusatsu, Shiga, Japan) at 37 °C for 15 min, followed by inactivation at 85 °C for 5 s and incubation at 4 °C. Real-time PCR was performed on a StepOnePlus real-time PCR system (Applied Biosystems, Tokyo, Japan), using Power SYBR Green PCR Master Mix (Applied Biosystems). The PCR conditions were as follows: 95 °C for 10 min, followed by 40 cycles of 95 °C for 15 s and 60 °C for 1 min. Real-time PCR was performed in triplicate. These data were analyzed by the ddCt method. For the quantitation of HAV RNA or β-actin mRNA, the following oligo primers were used: sense primer 5′-AGGCTACGGGTGAAACCTCTTAG-3′ and antisense primer 5′-GCCGCTGTTACCCTATCCAA-3′ for the former and sense primer 5′-CAGCCATGTACGTTGCTATCCAGG-3′ and antisense primer 5′-AGGTCCAGACGCAGGATGGCATG-3′ for the latter [15].

### 2.10. Statistical Analysis

Data are expressed as the means ± standard deviations (SDs). Statistical analyses were performed with Student’s *t*-test. *p*-values < 0.05 were considered significant. All assays were performed in at least triplicate. Statistical analysis was performed using DA Stats software (O. Nagata, Nifty Serve: PAF01644).

## 3. Results

### 3.1. Docking of the Selected Compound Series

The ten prioritized compounds (Figure 2) were docked into the catalytic region of the HAV 3C protease under identical conditions, and all compounds were accommodated within the targeted site. The best predicted Vina scores ranged from −7.612 to −5.752 kcal mol^−1^ (Table 1). Molecular weights ranged from 253.35 to 413.42 g mol^−1^, and the compounds contained 18 to 27 heavy atoms. The calculated physicochemical properties indicated general consistency with conventional Lipinski thresholds; however, **KCN-A-13** exhibited a cLogP of 5.59, which is slightly above the conventional lipophilicity threshold of 5. The ligand efficiency (LE) values clustered within a relatively narrow range of 0.247 to 0.328 kcal mol^−1^ per heavy atom. No direct correlation was observed between the predicted docking scores and antiviral activity. Notably, **KCN-A-12** demonstrated the strongest anti-HAV activity despite a comparatively modest Vina score of −5.835 kcal mol^−1^. These findings support the use of docking primarily to evaluate plausible active-site binding modes rather than to quantitatively predict cellular antiviral potency.

### 3.2. Effects of 10 Compounds on Survival of Huh7 Cells

**Figure 3:**
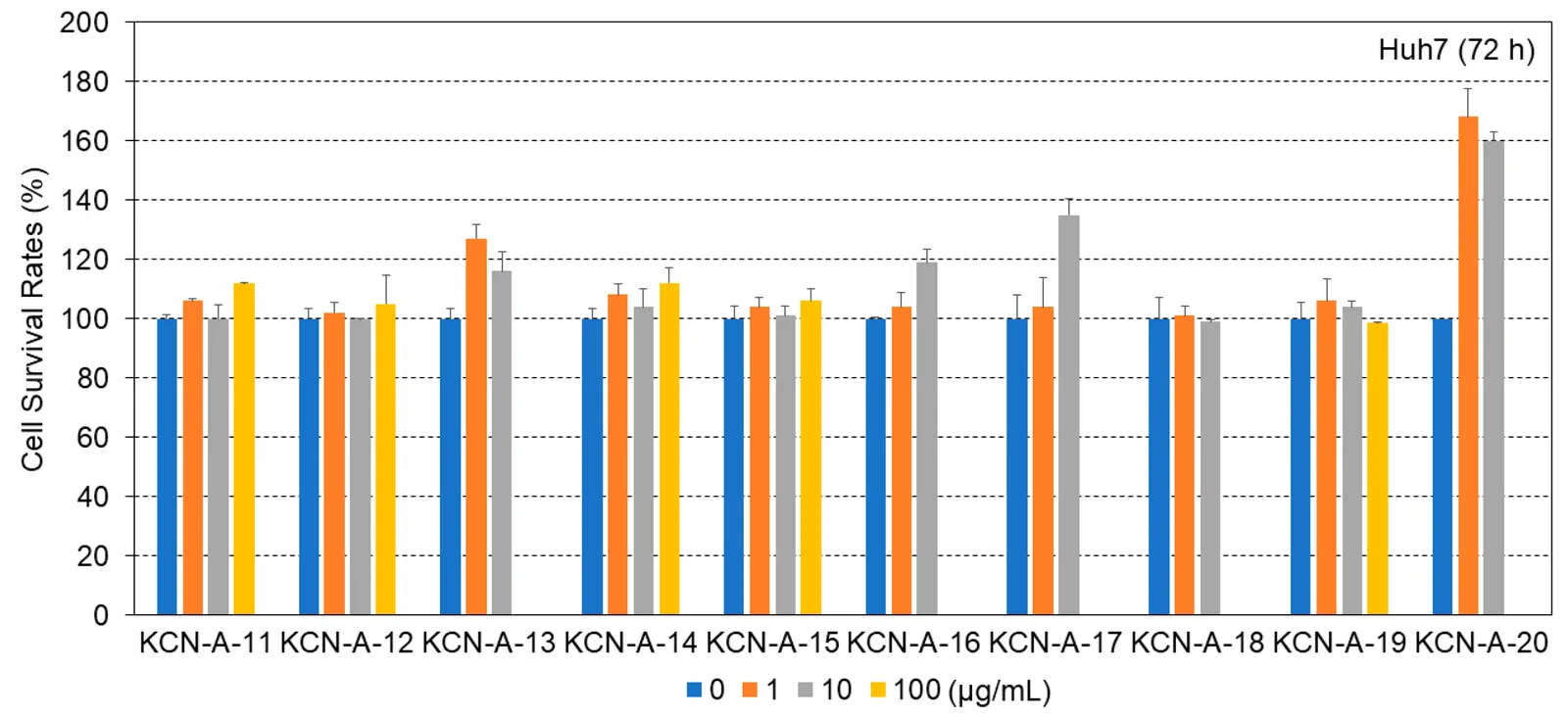
Effects of 10 compounds on cell viabilities of Huh7 cells. Huh7 cells were incubated with each compound for 72 h. Cell viabilities were determined via dimethylthiazol carboxymethox-yphenyl sulfophenyl tetrazolium (MTS) assay. Data are expressed as means and standard deviations of triplicate determinations from three independent experiments. Statistical significance was analyzed using the two-tailed Student’s *t*-test.

First, before we examined the effects of these 10 compounds on HAV replication in human hepatoma Huh7 cells, we studied their effects on the induction of cell death in Huh7 cells. The sensitivities of Huh7 cells to these 10 compounds were monitored for 72 h. It was shown that 100 μg of five compounds (**KCN-A-13**, **KCN-A-16**, **KCN-A-17**, **KCN-A-18**, and **KCN-A-20**) markedly reduced Huh7 cell viability, and that 100 μg of the other five compounds (**KCN-A-11**, **KCN-A-12**, **KCN-A-14**, **KCN-A-15**, and **KCN-A-19**) did not increase susceptibility in Huh7 cells (Figure 3). The half-maximal inhibitory concentration of five compounds (**KCN-A-13**, **KCN-A-16**, **KCN-A-17**, **KCN-A-18**, and **KCN-A-20**) on the Huh7 cells was < 100 μg/mL; and that of the other five compounds (**KCN-A-11**, **KCN-A-12**, **KCN-A-14**, **KCN-A-15**, and **KCN-A-19**) was >100 μg/mL. Therefore, we chose a concentration of 100 μg of these five compounds for experiments to screen the inhibition of HAV replication.

### 3.3. Effects of Five Compounds on HAV Genotype IB HM175/18f Replication in Huh7 Cells

**Figure 4:**
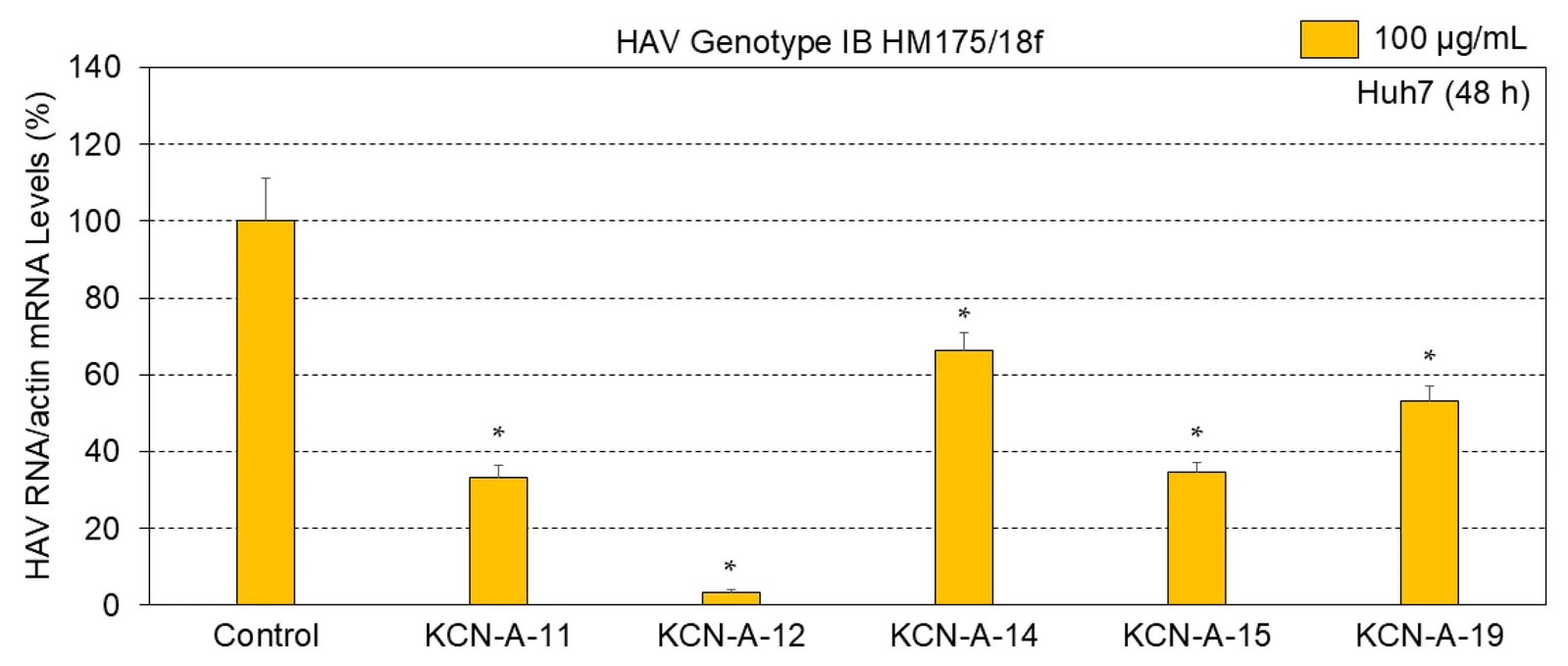
Effects of five compounds on HAV genotype IB HM175/18f replication in Huh7 cells. Cells were seeded before 24 h of HAV genotype IB HM175/18f infection at 0.01 MOI. After 24 h of infection, cells were incubated with each compound at 100 μg/mL for 48 h. Cellular RNA was collected at 72 h after infection, and HAV RNA/actin mRNA levels were measured by real-time RT-PCR. Data are expressed as means and standard deviations of triplicate determinations from three independent experiments. Statistical significance was analyzed using the two-tailed Student’s *t*-test. * *p* < 0.05.

After 24 h of HAV genotype IB HM175/18f infection, Huh7 cells were incubated with 100 μg of each compound—**KCN-A-11**, **KCN-A-12**, **KCN-A-14**, **KCN-A-15**, or **KCN-A-19**—for 48 h. After 72 h of HAV genotype IB HM175/18f infection, cellular RNA was extracted, and HAV RNA levels were determined using real-time RT-PCR. Five compounds (**KCN-A-11**, **KCN-A-12**, **KCN-A-14**, **KCN-A-15**, and **KCN-A-19**) could significantly inhibit HAV genotype IB HM175/18f replication (each: *n* = 3, *p* < 0.05). Among these five compounds, **KCN-A-12** treatment resulted in 3.4% of HAV genotype IB HM175/18f replication in Huh7 cells treated with the control (Figure 4). The IC_50_ of **KCN-A-12** is 37.5 μg/mL.

When the selectivity index (SI) was calculated for each compound using the formula of the half-maximal inhibitory concentration of each compound on the Huh7 cells/IC_50_ of each compound for HAV genotype IB HM175/18f replication, all five compounds (**KCN-A-11**, **KCN-A-12**, **KCN-A-14**, **KCN-A-15**, or **KCN-A-19**) showed greater efficacy against HAV genotype IB HM175/18f replication than the cytotoxicity against Huh7 cells (favorable SI > 1.0) (Figure 3 and Figure 4).

### 3.4. Effects of ***KCN-A-12*** on HAV Genotype IIIA HA11-1299 Replication in Huh7 Cells

**Figure 5:**
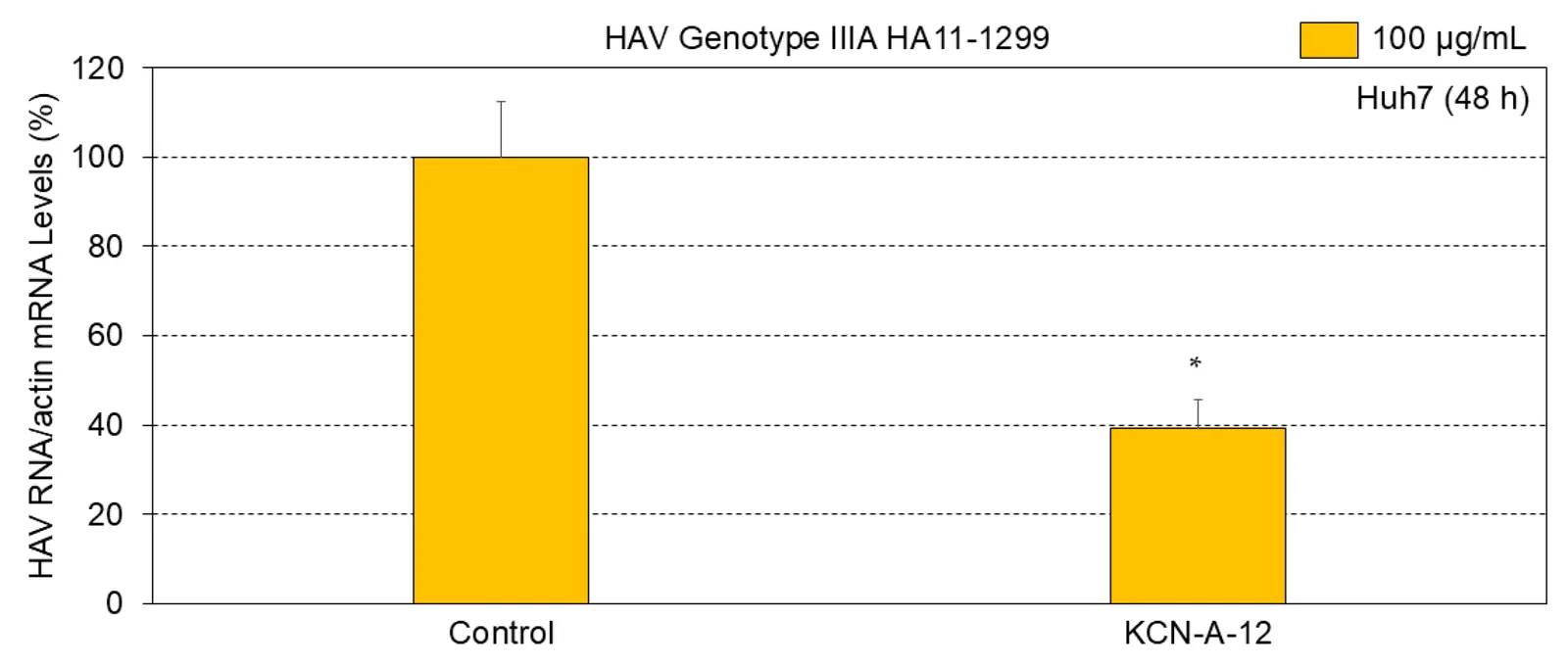
Effects of **KCN-A-12** on HAV genotype IIIA HA11-1299 replication in Huh7 cells. Cells were seeded before 24 h of HAV genotype IIIA HA11-1299 infection at 0.01 MOI. After 24 h of infection, cells were incubated with each compound at 100 μg/mL for 48 h. Cellular RNA was collected at 72 h after infection, and HAV RNA/actin mRNA levels were measured by real-time RT-PCR. Data are expressed as means and standard deviations of triplicate determinations from three independent experiments. Statistical significance was analyzed using the two-tailed Student’s *t*-test. * *p* < 0.05.

We next examined the effects of **KCN-A-12** on HAV genotype IIIA HA11-1299 replication in Huh7 cells. Similarly, after 24 h of HAV genotype IIIA HA11-1299 infection, Huh7 cells were incubated with 100 μg of **KCN-A-12** for 48 h. After 72 h of HAV genotype IIIA HA11-1299 infection, cellular RNA was extracted, and HAV RNA levels were determined using real-time RT-PCR. **KCN-A-12** was able to significantly inhibit HAV genotype IIIA HA11-1299 replication compared to the control (Figure 5). The **KCN-A-12** with efficiency against HAV genotype IIIA HA11-1299 replication greater than the cytotoxicity against Huh7 cells (favorable SI > 1.0) was indicated (Figure 3 and Figure 5). Thus, **KCN-A-12** significantly inhibited the replication of both HAV genotype IB HM175/18f and HAV genotype IIIA HA11-1299 in Huh7 cells.

### 3.5. Predicted Binding Mode of the Lead Compound ***KCN-A-12***

**Figure 6:**
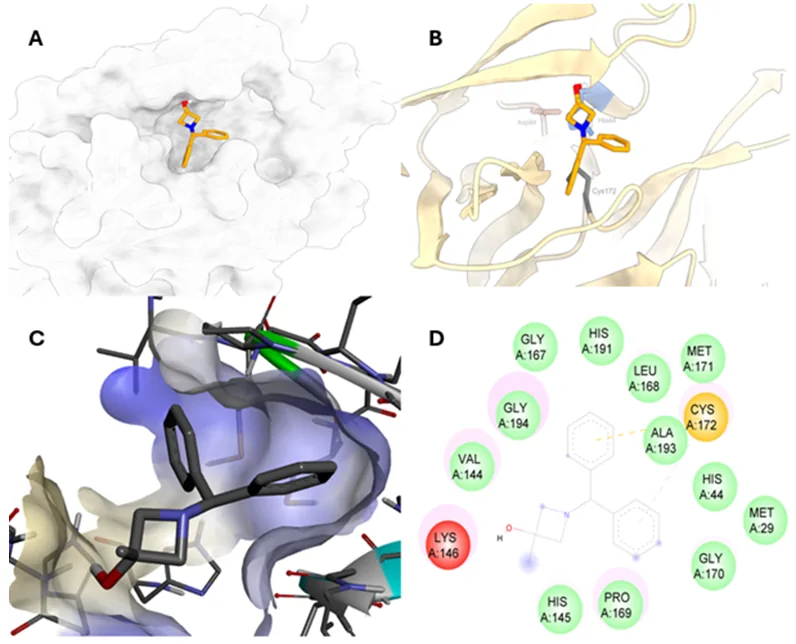
Predicted binding mode of **KCN-A-12** within the HAV 3C protease active site. (**A**) Surface view of **KCN-A-12** in the substrate-binding pocket. (**B**) Ribbon view showing its orientation relative to His44, Asp84, and Cys172. (**C**) Close-up view of **KCN-A-12** within the active-site cavity. (**D**) Two-dimensional interaction map showing catalytic and surrounding pocket residues.

Prediction models binding between **KCN-A-12** and the HAV 3C protease active site are shown in Figure 6. **KCN-A-12**, which exhibited the strongest anti-HAV activity, was predicted to bind within the proteolytic groove rather than at a peripheral surface site (Figure 6A,C). The ligand occupies a substantial proportion of the substrate-binding cavity, indicating favorable shape complementarity, and is positioned adjacent to the catalytic residues. One aromatic ring is oriented towards Cys172 and His44 (Figure 6B).

Interaction analysis identified a predicted π–sulfur contact between an aromatic ring of **KCN-A-12** and the sulfur atom of the catalytic Cys172. Additional hydrophobic and π–alkyl contacts involve Val144, Lys146, Pro169, Ala193, and Gly194. The binding site is further lined by van der Waals contacts with Met29, His44, His145, Gly167, Leu168, Gly170, Met171, and His191 (Figure 6D). Contacts extending towards the Gly170–Met171–Cys172 segment position the ligand near the oxyanion-hole region. The proximity to His191 orients part of the scaffold towards the S1 specificity pocket, which is associated with the enzyme’s preference for glutamine at the P1 position.

The predicted pose is dominated by hydrophobic and van der Waals contacts. No conventional hydrogen bond is formed between the azetidinol hydroxyl group and active-site residues in the selected pose. Overall, the predicted binding mode is consistent with steric occlusion of the substrate-binding groove and interference with substrate access to the catalytic machinery.

## 4. Discussion

HAV infections are still associated with morbidity and mortality, despite the fact that HAV vaccines are widely administered for their prevention [5]. To identify novel inhibitors of HAV 3C protease [18,19,20], an in silico study was performed, and its anti-HAV efficacies were examined in vitro. First, after the examination of the cytotoxicity of Huh7 cells, 5 out of 10 compounds were selected. Second, after examining the effects on HAV genotype IB HM175/18f replication in Huh7 cells, among the five compounds, **KCN-A-12** was identified as having an inhibitory effect < 10%. Third, after examining the effects on HAV genotype IIIA HA11-1299 replication in Huh7 cells, we also identified **KCN-A-12** as having an inhibitory effect.

Because most of the crystal structures of HAV 3C protease were based on the HAV genotype IB HM175 strain [18,19,20,21], we initially examined the effects on HAV genotype IB HM175/18f replication in Huh7 cells. Compared to the control (100%), a treatment of 100 μg/mL **KCN-A-12** for 48 h reduced HAV genotype IB HM175/18f replication in Huh7 cells to 3.4%. This suggests that **KCN-A-12** had a stronger inhibitory effect on HAV genotype IB HM175/18f replication in Huh7 cells.

A previous study demonstrated that Z10325150 could inhibit HAV genotype IIIA HA11-1299 replication [8]. Compared to the control (100%), a treatment of 100 μg/mL Z10325150 for 72 h reduced HAV genotype IIIA HA11-1299 replication to 69.6% [8]. In the present study, compared to the control (100%), a treatment of 100 μg/mL **KCN-A-12** for 48 h reduced HAV genotype IIIA HA11-1299 replication in Huh7 cells to 39.3%, suggesting that **KCN-A-12** seems to have an inhibitory effect on HAV genotype IIIA HA11-1299 replication in Huh7 cells.

Of interest, it seems that **KCN-A-12** has a stronger inhibitory effect on the replication of HAV genotype IB HM175/18f than that of HAV genotype IIIA HA11-1299 in Huh7 cells. The nucleotide length of the HAV 3C coding region from HAV genotype IB HM175/18f [22] is shorter than that from HAV genotype IIIA HA11-1299 (660 nt. vs. 657 nt.). Compared nucleotide substitutions between HAV genotype IB HM175/18f and HAV genotype IIIA HA11-1299, those are 16% (105/657) and 15.9% (105/660). Thus, as a difference in nucleotide sequences of the HAV 3C protease coding region exists between the two strains, structural comparison of HAV 3C proteases will be needed between HAV genotype IB HM175/18f and HAV genotype IIIA HA11-1299.

The primary screening experiments were performed at 100 μg/mL. This appears to be a relatively high concentration. The 100 μg/mL of **KCN-A-11**, **KCN-A-12**, **KCN-A-13**, **KCN-A-14**, **KCN-A-15**, **KCN-A-16**, **KCN-A-17**, **KCN-A-18**, **KCN-A-19**, and **KCN-A-20** are equal to 336 μM, 394 μM, 349 μM, 385 μM, 379 μM, 350 μM, 241 μM, 282 μM, 335 μM, and 291 μM, respectively. The IC_50_ of **KCN-A-12** is 148 μM.

MTS assays might measure metabolic activity rather than cell death directly. When trypan blue dye exclusion assays were also performed [16], the viabilities of Huh7 cells were 95.3 ± 0.5, 95.3 ± 0.5, 94 ± 1.4, 94.6 ± 0.5, 82.3 ± 2.0, and 45.3 ± 5.2 after 72 h of treatment of **KCN-A-12** at 0, 1, 10, 100, 150, and 200 μg/mL, respectively. The 1–100 μg/mL **KCN-A-12** did not significantly reduce the viabilities of Huh7 cells. The half-maximal inhibitory concentration of **KCN-A-12** on the Huh7 cells was 192 μg/mL (759 μM). When the SI was calculated for each compound using the formula for the half-maximal inhibitory concentration of **KCN-A-12** on the Huh7 cells by trypan blue dye exclusion assay/IC_50_ of **KCN-A-12** for HAV genotype IB HM175/18f replication, **KCN-A-12** also indicated a drug with greater efficacy against HAV genotype IB HM175/18f replication than the cytotoxicity against Huh7 cells (favorable SI 5.12).

Immunity or vaccination against HAV infection is useful as pre-exposure prophylaxis (PrEP) [23]. Postexposure prophylaxis (PEP) with hepatitis A vaccine or immune globulin effectively prevents HAV infection within 2 weeks of exposure [24]. There are no anti-HAV drugs that are useful for PrEP or PEP for HAV infection. From the viewpoint of public health, drug discovery in virology, including in HAV infection, is important [1].

The process of drug discovery for HAV infection starts with the identification of the target (such as HAV 3C protease), followed by target validation, hit discovery, lead optimization, and preclinical/clinical development [25]. In the present study, we targeted HAV 3C protease inhibitors, identified several drugs by in silico screening, and finally found an effective compound, **KCN-A-12**, which had a stronger inhibitory effect on both HAV genotype IB HM175/18f and HAV genotype IIIA HA11-1299 replication in vitro.

There are several limitations in the present study: (1) we may not measure the reduction in HAV productive replication. Orthogonal validation, such as measurement of extracellular viral RNA/infectious virus and viral protein expression, may be needed; (2) the impact of compounds on viral replication under varying MOI conditions should be assessed; (3) crystal structures of HAV 3C protease based on the HAV genotype IB HM175 strain were used for the in silico study—we may use those derived from other HAV strains. Structural comparison of HAV 3C proteases between HAV genotype IB and HAV genotype IIIA should be needed; (4) our study of **KCN-A-12** lacks protease assay, molecular dynamics analysis or in vivo validation, although protease assay may not always be needed [26]; (5) their safety and effects could be confirmed by an in vivo study; and (6) compound **KCN-A-12** could be further modified to develop more effective drugs with fewer adverse events.

## 5. Conclusions

We evaluated more effective HAV 3C protease inhibitors with activity against both HAV genotype IB HM175/18f and HAV genotype IIIA HA11-1299-infected human hepatoma cell lines. In particular, we showed that **KCN-A-12** suppressed HAV replication and is predicted to inhibit the protease activity. In silico screening for HAV 3C protease inhibitors may be useful for further discovery of anti-HAV drugs.

## Figures and Tables

**Table 1:** Molecular formulas, physicochemical properties, best AutoDock Vina scores, and ligand efficiencies for compounds **KCN-A-11** to **KCN-A-20** docked against the HAV 3C protease.

Compound	Molecular Formula	Molecular Weights	Heavy Atoms (*N*) ^a^	Vina Score (kcal mol^−1^)	Ligand Efficiency (*LE*) ^b^
**KCN-A-11**	C_18_H_19_NO_3_	297.35	22	−6.825	0.310
**KCN-A-12**	C_17_H_19_NO	253.35	19	−5.835	0.307
**KCN-A-13**	C_17_H_23_BO_3_	286.18	21	−6.809	0.324
**KCN-A-14**	C_12_H_21_NO_5_	259.30	18	−5.752	0.320
**KCN-A-15**	C_14_H_21_N_3_S	263.40	18	−5.907	0.328
**KCN-A-16**	C_12_H_13_F_2_N_3_OS	285.31	19	−5.938	0.313
**KCN-A-17**	C_17_H_21_F_6_N_3_S	413.42	27	−7.612	0.282
**KCN-A-18**	C_18_H_28_N_2_O_5_	354.45	25	−6.169	0.247
**KCN-A-19**	C_17_H_18_N_2_O_3_	298.34	22	−6.686	0.304
**KCN-A-20**	C_20_H_29_N_3_O_2_	343.47	25	−6.840	0.274

^a^ N = number of non-hydrogen (heavy) atoms. ^b^ Ligand efficiency, LE = −S/N, reported in kcal mol^−1^ per heavy atom, S = docking score.

## Data Availability

The original contributions presented in this study are included in the article. Further inquiries can be directed to the corresponding authors.

## Abbreviations

The following abbreviations are used in this manuscript:

HAV	Hepatitis A virus
MSM	Men who have sex with men
KCDC	Korean Centers for Disease Control and Prevention
RPMI	Roswell Park Memorial Institute medium
FBS	Fetal bovine serum
PBS	Phosphate-buffered saline
MOI	Multiplicity of infection
MTS	Dimethylthiazol carboxymethoxyphenyl sulfophenyl tetrazolium
IC_50_	Half-maximal inhibitory concentration
SDs	Standard deviations
PrEP	Pre-exposure prophylaxis
PEP	Postexposure prophylaxis
